# Comparing Self-Report vs. Performance Measures of Attentional Control and Efficiency

**DOI:** 10.3390/neurosci5020008

**Published:** 2024-04-04

**Authors:** Mohammad Ahsan Khodami, Luca Battaglini, Maryam Jansarvatan, Sofia Kireeva, Seiran Bagheri

**Affiliations:** 1 Department of General Psychology, University of Padua, 35131 Padua, Italy; 2 Graduate School of Economics and Management, Ural Federal University, 62002 Ekaterinburg, Russia; 3 Departmen of Psychology, Payame Noor University, Tehran 19395-4697, Iran

**Keywords:** attention, attention control, attentional control scale, attention network test, psychometric

## Abstract

**Background:** The Attention Control Scale (ATTC) is a widely used self-report measure of attentional control capacities. However, research questions whether it accurately substitutes for objective attention control tasks. This study investigated ATTC’s correlation with the Attention Network Test (ANT) across alerting, orienting, and executive control networks. We also used the Inverse Efficiency Score (IES) as an additional factor to check ATTC using ANT. **Methods:** We administered 143 participants who completed the ATTC questionnaire and ANT behavioral test assessing network efficiencies. **Results:** The results showed non-significant ATTC-ANT correlations across all networks. In an additional analysis, while the ATTC demonstrated factorial validity, subjective control was disconnected from actual attention regulation efficiency. A small male advantage emerged for executive control. **Conclusions:** Dissociations likely stem from attention complexity and method variances rather than overlap. The findings do not support the ATTC as a stand-alone proxy for performance-based measurement. Multifaceted assessments are essential for comprehensively capturing attentional control.

## 1. Introduction

Human behavior and cognition emerge from complex interactions between multiple mental processes. Attention represents a core capacity that selects, maintains, and shifts focus among all the stimuli bombarding our senses [1]. Attention control is the cognitive process of regulating attention through monitoring, selectivity, and flexibility. This multidimensional construct consists of specific attentional networks like alerting, orienting, and executive control [2]. Attention also allows priming relevant information while suppressing irrelevant noise. This filtering mechanism is essential for higher-order functions like learning, memory, reasoning, and decision-making to operate on a manageable subset of inputs [3]. While attention can be reflexively captured by salient stimuli, voluntary attentional control provides additional flexibility to direct focus based on current goals, expectations, and knowledge [4].

Attention control is the principal cognitive construct regulating various sub-cortical and cortical processes that effectively select and filter sensory information [5]. These interrelated sub-processes notably include the top-down modulation of attentional orientation reflecting current goals, the active inhibition of external and internal distractions, rapid flexibility in allocating attention between competing stimuli and changing situational demands, and continuous monitoring and timed updating of working memory representations [6]. Interpreting the precise neurocognitive mechanisms mediating these sub-processes and their dynamic integration remains an active area of scientific inquiry [7].

Attentional control abilities vary across individuals and relate to real-world outcomes. For example, deficits in controlling attention have been associated with clinical conditions like ADHD, anxiety, and depression [8,9]. Enhancing attentional control through training can improve cognitive performance and reduce symptomatology [10]. Given the importance of attentional control, accurately measuring it has implications for basic science and applied settings. However, assessment is complicated by the multifaceted nature of attention [11]. Focusing, shifting, and executive control represent different components that may not behave uniformly.

Questionnaire methods offer one approach to assess attentional control. Among them, the Attention Control Scale (ATTC or ACS) is a widely used self-report measure requiring individuals to place their capacity to focus and shift attention flexibly [12]. While convenient and easy to administer, this questionnaire is popular and widely used in studies (for review, see [13]). Some work has questioned the reliability of ATTC. Some studies show congruent relationships, while others do not [14,15], suggesting that establishing the convergence between subjective and objective attentional control measures has important theoretical and practical implications. A meta-analysis by [13] showed that the ATTC is not significantly associated with objective measures of attention control, suggesting that it may not accurately reflect attention control capacities.

Emerging computer-based assessments provide an alternative approach to objectively evaluating attentional control. Integrating subjective questionnaires and objective computer-based tasks could provide complementary insight into attentional control and its neural and cognitive mechanisms. Assessing questionnaires alongside behavioral and computerized tasks is vital for thoroughly understanding human behaviors and cognitive processes [16].

Questionnaires provide subjective self-reports, capturing individuals’ perceptions, attitudes, and self-assessments of behaviors, which are invaluable for understanding psychological states and traits. However, they can be limited by biases such as social desirability and self-awareness [17,18]. Behavioral and computerized tasks complement this by objectively measuring performance and responses on controlled grounds. These tasks can demonstrate cognitive and behavioral patterns that must be evident or accurately reported in questionnaires. By combining both methods, researchers can acquire a more potent and holistic perspective, enhancing the validity and reliability of their findings in psychological and cognitive research.

The current study investigates whether the ATTC can measure the same aspects observable through behavioral tasks. The primary objective was to determine whether ATTC is a dependable method for assessing attentional control or, as suggested by [13], if it lacks validity. Given the impracticality of employing every available task, the Attentional Network Task (ANT) was selected to examine the ATTC Scale.

Although some research in this area has been carried out, most of these studies focused on anxiety and mood as factors. Also, studies in this area were mostly carried out via presentation in the laboratory or via presence; the author of [19] further emphasizes this point, noting that the artificial environment of the lab can warp effect sizes, making them less applicable. Also, the author of [20] adds a phenomenological perspective, arguing that reducing problem-solving behavior to a cognitive mechanism in the lab overlooks important experiential aspects. Considering most of these findings, we have decided to administer this task in an online environment, which is less stressful and more based on the participants’ favorite time to do the task.

The Attentional Network Task (ANT) is used in cognitive psychology, particularly in studying attention. While there is limited direct information on using ANT to validate ATTC specifically, we can infer some reasons based on their functions and characteristics. First, it is a self-report measure that assesses individual differences in attentional control, focusing on the ability to maintain and shift attention between tasks [21]. On the other hand, ANT is a performance-based task that measures the efficiency of different attention networks (alerting, orienting, and executive control) in the brain. Using ANT to validate ATTC can be advantageous as it complements self-reported data with objective performance-based data, providing a more holistic view of an individual’s attentional control capacities [22,23].

In some studies involving functional magnetic resonance imaging (fMRI), ANT validated ATTC [24]. This suggests that ANT can be effectively used in different settings to validate the ATTC, which is important for generalizing the findings to various contexts. Also, the authors of [22,23,25] mention that the ANT is a suitable choice for observing ATTC due to its ability to provide objective, performance-based data that complement the self-reported nature of ATTC, its coverage of a broad range of attentional processes, and its potential to demonstrate the neural correlates and broader implications of attentional control.

## 2. Materials and Methods

### 2.1. Procedure

#### 2.1.1. Participants

A power analysis was conducted using R Software [26] and the pwr package [27]. Before data collection to determine the required sample size for detecting medium correlations between the Attention Control Scale total and sub-scale scores (ATTC_TOT, ATTC_FOC, ATTC_SHI) and the Attention Network Test outcomes for the alerting, orienting, and executive control networks (alerting, orientation, and executive). The significance level was adjusted to 0.0056 (0.05/9) using a Bonferroni correction for the nine planned analyses to account for the multiple correlations. This adjustment ensures 80% power to detect an effect size of 0.30 for each test. The prior power analysis indicated that a sample size of 138 was required.

#### 2.1.2. Procedure

For this research, we emailed a list of students who registered on the voluntary system to participate in the LinguaMed learning group. Also, we provided QR codes, direct links, and short links in the group chats to recruit participants. Each participant was directed to complete a Machform (http://www.matchform.com (accessed on 1 January 2023)) survey hosted on a personal server, accessible via the link. To ensure a diverse representation, we limited participation to up to two users per platform, device, or IP address. If a participant attempted to open the survey link on a smaller screen, such as a mobile phone, they were automatically presented a message asking them to complete the survey on a computer screen instead.

Before proceeding, participants were required to read and either accept or decline a consent form. In our consent form, we asked our participants to confirm if they had any psychological diagnoses. Upon confirmation and receiving, they were directed to provide demographic information, including age, gender, and handiness. Subsequently, they completed the ATTC questionnaire. Following this, participants were redirected to the Pavlovia (https://pavlovia.org/ (accessed on 1 January 2023)) platform, where they engaged in the ANT task online. Participants first completed 15 ANT practice trials to familiarize themselves with the task requirements. This was followed by 120 ANT test trials assessing attentional network efficiencies through reaction time (RT) and accuracy indices. Participants could withdraw their participation through consent and study procedures; incomplete datasets were excluded from analysis.

Participants were informed beforehand that no prize, credit, or results would be provided at the task’s conclusion. Furthermore, they were advised of their right to withdraw from participation at any stage during both the questionnaire and the ANT task.

#### 2.1.3. Tasks

**Attention Control Scale ATTC**

The Attention Control Scale (ATTC) is a 20-item self-report psychometric instrument developed to assess perceived capacity for voluntary attention control across two key domains: attentional focusing and attentional shifting. The ATTC requires respondents to rate the typical frequency of experiences relating to concentrating, ignoring distractions, and flexibly directing focus using a 4-point Likert scale format (1 = almost never; 4 = always). Initial validation analyses indicated adequate internal reliability for the composite ATTC (α = 0.88) and respective Attentional Focusing (α = 0.80) and Attentional Shifting (α = 0.65) sub-scales [12]. Principal components analysis verified the two-factor structure, which cumulatively accounted for 29% of the variance. Test-retest reliability over a 2-week interval was also acceptable (*r* = 0.76). Higher ATTC scores theoretically index greater attention control.

**Attentional Network Task ANT**

The Attention Network Test (ANT) is a behavioral procedure developed by Fan and colleagues [25] to evaluate the efficiency of three attentional networks—alerting, orienting, and executive control—within a single integrated task. The task requires participants to indicate the direction of a central arrow target flanked by congruent, incongruent, or neutral arrows. Warning cues presented before target onset manipulate alertness and spatial attention. By comparing performance (reaction times, accuracy) across cue and flanker conditions via subtractions, the ANT provides indexes of alerting, orienting, and conflict resolution capacities, demonstrating moderate test-retest reliability. The ANT cue-flanker manipulations also interact, with alerting cues enhancing distractor interference, suggesting functional interactions between networks [25,28]. We reproduced the exact experiment as Fan et al. did (for review [25]).

## 3. Data Analysis

We used Python code to calculate the total score, attentional shift, and attentional focus based on the questionnaire detail provided [12]. Since the main package lmsupport had technical problems calculating varScore, we adopted the varScore code from the main package and reran it to calculate the Attentional focus, shift, and total attention score see **Appendix code** (https://osf.io/8ptx9 (accessed on 20 December 2023)). All statistics and analyses were carried out using Python and statistical packages.

Before parametric statistical analyses, the normality assumption was assessed using Shapiro–Wilk tests to assess the three attitude measures—ATTC_TOT, ATTC_FOC, and ATTC_SHI. For the total attitude score ATTC_TOT, the Shapiro–Wilk test produced a W statistic of 0.9901 and a non-significant p-value of 0.4103, indicating that the distribution of ATTC_TOT scores does not significantly deviate from a normal distribution. Similarly, for ATTC_FOC and ATTC_SHI, the Shapiro–Wilk tests yielded non-significant p-values of 0.07126 and 0.09507, respectively. As all *p*-values are greater than the 0.0056 significance level, the null hypothesis that the data are normally distributed cannot be rejected. Therefore, the assumption of normality was deemed to have been met for using these variables in parametric statistical tests.

## 4. Results

### 4.1. Demographic Detail

In this study, we recruited a cohort of 143 participants, comprising 71 females and 72 males, with an average age of 26.15 years (SD = 5.04). Eighty-four of the participants were right-handed. Notably, due to the lack of a finishing questionnaire or odd pattern of answering (e.g., all answers were a specific number or the task still needed to be finished), we eliminated 17 participants from our results to conduct analysis.

### 4.2. Attentional Questionnaire Scale Result

In our study, the Attentional Control Scale (ATTC) results for Total Attention (ATTC_TOT) had a mean score of 50.38 with a standard deviation of 4.63; the Focus sub-scale (ATTC_FOC) had a mean score of 22.74 with a standard deviation of 3.48; and, lastly, the Shift sub-scale (ATTC_SHI) had a mean score of 27.64 with a standard deviation of 3.48.

**Figure 1:**
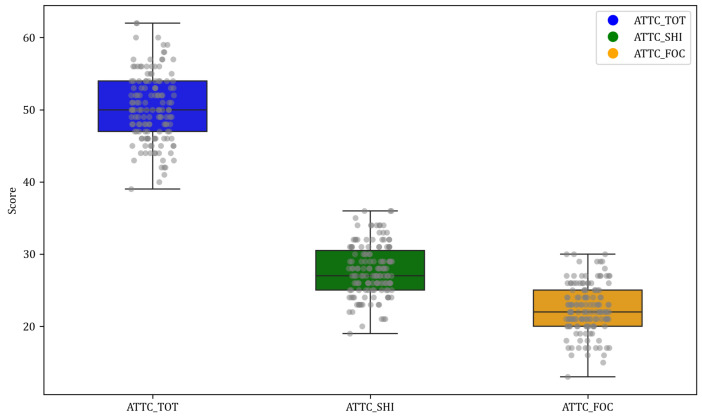
Box plot of measures for ATTC: *ATTC_TOT* represents the Attentional Control Scale Total; *ATTC_FOC* denotes the Attentional Control Scale Focus sub-scale; and *ATTC_SHI* signifies the Attentional Control Scale Shift sub-scale.

The results revealed no significant gender differences in focus scores (t(df)=1.18, p=0.24), total scores (t(df)=0.49,p=0.62), or shift scores (t(df)=−0.46,p=0.64). Furthermore, the small Cohen’s d effect sizes (range −0.08 to 0.20) and confidence intervals for mean differences containing zero (−1.45 to 1.77) indicated negligible differences between genders across all facets of attentional control. Table 1 shows the questionnaire results, and Figure 1 below illustrates the data distribution among participants for this task.

### 4.3. ANT Task Result

**Figure 2:**
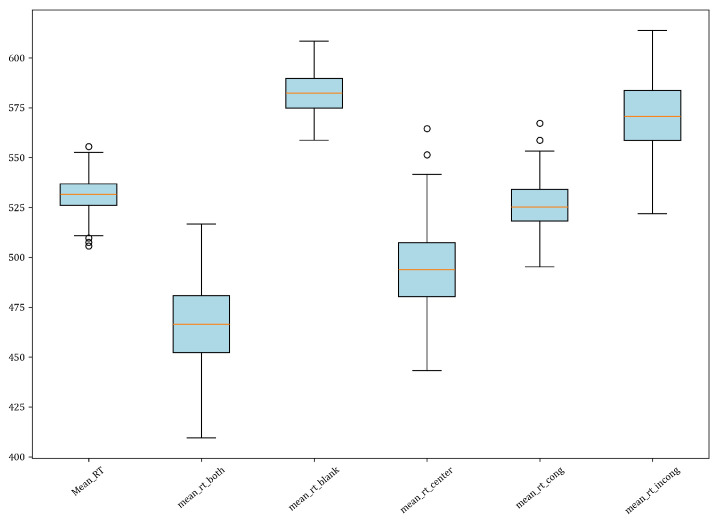
Box plot of response time (RT) for different conditions in the ANT task.

A descriptive analysis examined response time performance across the ANT conditions. Mean response times were faster for the double cue condition (M = 467.11, SD = 19.47) than the no cue condition (M = 582.03, SD = 10.31), indicating enhanced alerting with the availability of an alerting cue. Additionally, response times were slower for incongruent flanker trials (M = 570.57, SD = 18.23) than congruent trials (M = 525.81, SD = 12.95), suggesting greater executive control demands and conflict resolution for incongruent stimuli. Differences between other trial types were negligible. In sum, the expected effects of alerting and executive attention were evidenced in the descriptive results, whereby alerting cues sped responses through enhanced readiness, and incongruent conditions elicited responses to conflict and slower reactions. Figure 2 and Table 2 show a description of this analysis.

The current results demonstrate significant effects of cue type on reaction times, with the double “both” cue producing the fastest reaction times overall compared to the spatially neutral “center” and no cue "blank" conditions. This incremental speeding of reaction time suggests enhancement of alertness and spatial orienting when temporal and spatial cues are provided before the target.

**Figure 3:**
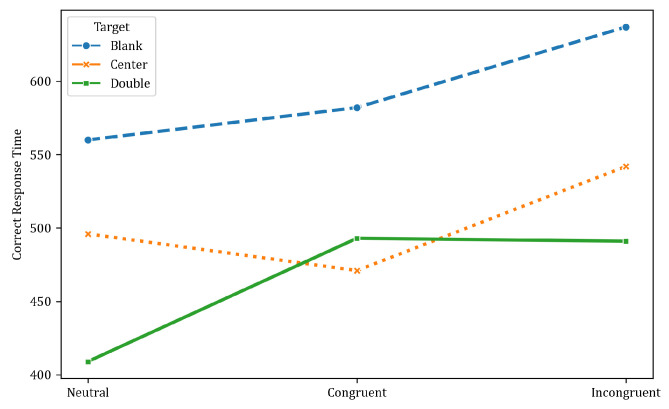
Reaction time “resp.rt” in ms for the ANT task based on the category of warnings provided to participants for ALL trials.

Additionally, as Table 3 and Figure 3 show, reaction times were slowest in response to incongruent stimuli relative to congruent and neutral target conditions. This pattern indicates that executive control processes are recruited more when conflicting stimulus-response mappings induce competition, reflecting enhanced demands on selective attention. Participants took longer to respond to incongruent stimuli for all cue types than congruent and neutral conditions. This demonstrates increased cognitive effort and reduced processing efficiency when the attentional focus was directed to overcome incongruence.

#### 4.3.1. Inverse Efficiency Score IES

The Inverse Efficiency Score (IES) is a widely used composite measure, originally proposed by [29]. It is generally calculated as the ratio of mean correct response times (RTs) to the proportion of correct responses (CRs), as defined by [29] and later by [30]:

(1) $$IES_{i,j}=\frac{\bar{RT_{i,j}}}{CR_{i,j}}$$

where $\bar{RT_{i,j}}$ represents the mean RT of participant *i* for correct responses in condition *j*, and $CR_{i,j}$ represents the proportion of correct responses of participant *i* in condition *j*. While the IES has mostly been calculated using only correct RTs in empirical studies, the authors of [31]’s original concept suggested considering all RTs, including those from erroneous trials. However, this analysis focuses on the more commonly reported version, which excludes incorrect RTs [32]. The IES is a valuable index in psychological research because it offers a holistic assessment of task performance efficiency, sensitive to individual differences, reflective of cognitive processing strategies, comparable across conditions, and supported by empirical evidence [33].

#### 4.3.2. Calculate ANT Sub-Scales

Data from the ANT test drive attention sub-scales. Based on [25]:

- **Alerting** is calculated by subtracting the mean RT of Both cue conditions from the mean of RT for the None Cue condition. This is because neither of these conditions provided information about the target or whether the stimulus would appear above or below the fixation point. In contrast, in the None condition, attention also remains diffused between it.
- **Orientation** is calculated by subtracting the RT mean for Both minus center; this is because, in both conditions, we have either the top or down in which, in this condition, cue serves as predictive spatial information that allows the subject to begin Orientation-appropriate locations before the target arrives.
- **Executive control** is calculated by subtracting all conditions where the cue is congruent from the total RT of incongruent data.

An independent samples *t*-test analyzed gender differences. As seen in Table 4, there were no significant differences between males and females on either alerting, t(df)=0.18, p=0.86, Cohens d = 0.03, 95% CI = [−6.53, 7.82], or orienting, t(df)=0.47, p=0.64, Cohens d = 0.08, 95% CI = [−6.69, 10.96]. However, a small-moderate difference emerged for executive control network efficiency, t(df)=−2.34, p=0.02, Cohens d = −0.39, 95% CI = [−14.99, −1.33], with males demonstrating significantly enhanced executive functioning compared to females. While alerting and orienting performance was equivalent across genders, males showed an advantage in executive attentional control. While small in magnitude, this effect may have implications for males’ superior regulation of cognition and behaviors relative to females [34].

#### 4.3.3. Correlational Analysis

The correlation analysis revealed varying degrees of association among the study variables, with ATTC_FOC showing a strong positive correlation with ATTC_TOT (*r* = 0.65, *p* < 0.001) and other notable correlations, as detailed in Table 5.

**Figure 4:**
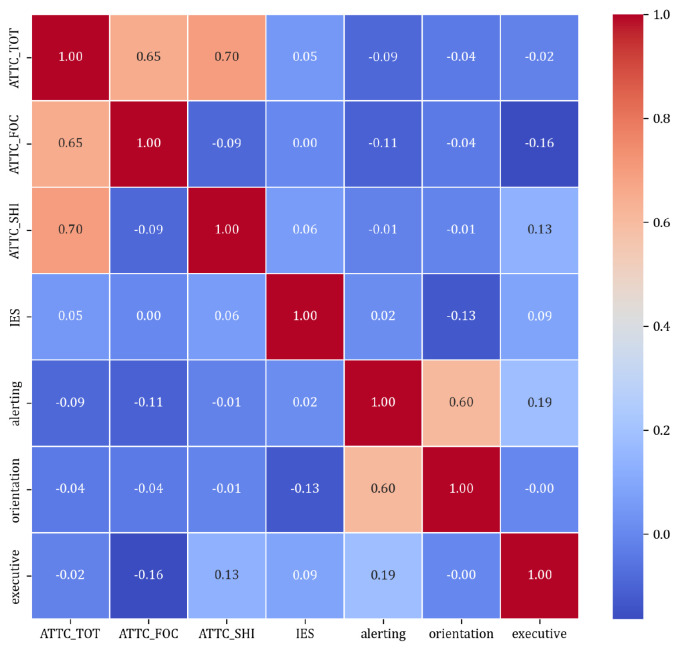
The correlation map between the ATTC scores (ATTC_TOT, ATTC_FOC, ATTC_SHI) and the ANT outcomes (alerting, orientation, executive, and IES).

Figure 4 shows the correlation mconsidered variables study, which does not suggest any strong correlation between our considered variables.

Also, for IES, we could not find any correlational significance suggesting that data obtained from the ATTC questionnaire cannot predict the outcomes of the ANT task.

## 5. Additional Analysis

After finishing our analysis, we decided to conduct another additional analysis. This analysis aimed to check some other aspects of our data, so a confirmatory factor analysis (CFA) was conducted on the Attention Control Scale (ATTC) items to validate the hypothesized two-factor structure measuring attentional focus and shifting ability.

The CFA supported the expected two-factor model ($\chi^{2}\left(0\right)=0.00,\;p>0.05$) with fit based on standard criteria (CFI = 1.00, TLI =1.00, RMSEA = 0.00, and SRMR = 0.00). All ATTC items loaded significantly onto their specified factors (p<0.001). The “focus” factor consisted of three items related to one’s capacity to intentionally maintain attentional focus (std. loadings 0.33–0.41). The “shifting” factor contained two items measuring ease of switching focus (std. loadings 0.35–0.36). The factors showed a small, non-significant negative correlation (r=−0.09, p=0.30).

Even with the Attentional Control Scale (ATTC) showing solid factorial validity in the confirmatory factor analysis, we still do not see significant correlations between the ATTC scores and Attention Network Test (ANT) performance.

Research on the correlation between psychological questionnaires and behavior reveals mixed findings. Ref. [35] suggests that certain psychological features, such as a sense of being undervalued, can be linked to specific behaviors. However, ref. [36] warns that people tend to overestimate the stability of behavior across circumstances, leading to inaccurate correlations. Ref. [37] further problematizes the issue by finding that the Questionnaire for the Measurement of Psychological Reactance, a widely used tool, provides unreliable estimates. Ref. [38] adds another layer of complexity, highlighting the potential for measurement operations to influence correlations among measures. These studies collectively suggest that while psychological questionnaires may offer some insight into behavior, their accuracy and reliability are not guaranteed.

We also ran a multiple regression study to check if we could find any correlation between factors or not; based on the multiple regression results, we could not see any significant relationships between attentional control as measured by the ATTC factor scores and performance on the objective ANT as assessed by reaction time metrics of the alerting, orienting, or executive control networks. Specifically:

In the model predicting alerting network efficiency (alerting reaction time), neither the ATTC focus nor shifting coefficients were significant predictors (ATTC_FOC: b=−0.699, p=0.20; ATTC_SHI: b=−0.143, p=0.782).

Similarly, for the orienting network model, the ATTC factors did not significantly predict orienting efficiency scores (ATTC_FOC: b=−0.319, p=0.636; ATTC_SHI: b=−0.115, p=0.858). In the executive control model, while the ATTC focus factor was close to the significance level (b=−0.956, p=0.068), overall, the subjective attention measures did not reach significance for explaining executive network reaction times either. So, in all three attentional domains, an individual’s attentional control showed little connection to how efficiently they completed the ANT behavioral tasks engaging those specific control processes.

This aligns with the lack of previous Pearson correlations between the ATTC and ANT metrics. The reasons above, like differences between explicit perceptions versus implicit behaviors, likely explain the continued lack of significant relationships in the regression models.

## 6. Discussion

The current study tried to connect self-reported attentional control on the Attentional Control Scale (ATTC) and performance-based attentional control efficiency assessed by the Attention Network Test (ANT). However, the results did not support any connection, revealing non-significant relationships between ATTC scores and ANT outcomes across alerting, orienting, and executive control networks. This finding is consistent with [13], who reported that ATTC scores do not reliably predict objective attention control, suggesting the ATTC measures subjective perceptions rather than actual control abilities. Our study further questions the validity of the ATTC as a proxy for attentional control efficiency.

This dissociation between subjective self-reports and objective behavioral measures aligns with theoretical mo dels proposing distinct pathways for explicit, conscious self-evaluation versus implicit cognitive processing and performance [39,40]. While self-report scales like the ATTC assess a person’s explicit beliefs about their attentional capacities, these metacognitive judgments may not accurately track the implicit allocation of attentional resources during demanding cognitive tasks like the ANT. This divergence could stem from limitations in self-awareness, whereby individuals have incomplete introspective access to their own attentional control abilities.

Despite these results, the ATTC exhibited good factorial validity in a confirmatory analysis, indicating it reliably measures respondents’ perceived focusing and shifting capacities. Nevertheless, this insight seems disconnected from actual attention regulation in behavioral tasks. This dissociation could be due to the multidimensional nature of attention, as discussed by [41], making it challenging for a single task like the ANT to capture attention’s complexity fully. Moreover, inherent variances between self-report methods, computerized testing, and real-world functioning [42] may contribute to this discrepancy, compounded by limitations in self-awareness.

The present findings raise broader questions about how self-report and behavioral measures of cognitive processes will converge or diverge. Existing theories propose that subjective self-reports index stable trait-level characteristics better, while objective task performance is more sensitive to state-level fluctuations [43,44]. From this perspective, the ATTC may capture trait-level beliefs about attentional control abilities that remain stable across situations, whereas performance on the ANT is more variable and context-dependent. This could explain the observed disconnect, with implications for when each type of measure is more appropriate for assessment.

Furthermore, methodological differences between self-report and objective tests warrant consideration. Personal biases often influence subjective measures, while performance-based tests offer a more controlled evaluation environment. Investigating the neurocognitive support of attention control could clarify the discrepancies between these measures as the brain regions and networks involved may respond differently in subjective versus objective assessments.

Considering behavioral data and questionnaires, behavioral and psychological questionnaires often do not align due to various factors. Ref. [45] highlights the limitations of behavioral data in capturing the psychological processes that underpin group behavior. Ref. [46] further emphasizes this point, showing a modest overlap between psychometric and self-reported definitions of executive function deficits in individuals with ADHD.

Individual differences also play a critical role [9,47,48]. Variability in personality traits; cognitive abilities, especially in attention in different tasks [49,50,51]; and mental health status might influence the relationship between self-reported and objectively measured attention control. This aspect underscores the importance of considering a broad range of individual characteristics in future research.

Given these complexities, our findings do not support using the ATTC as a sole measure for attention control efficiency. Instead, a more nuanced approach is required, integrating various methods and perspectives. This includes longitudinal studies to track changes in attention control over time and alternative measurement instances encompassing broader psychological constructs. Such multifaceted approaches are essential for comprehensively understanding attention control and its assessment.

## 7. Conclusions

The current study found no significant correlation between the Attentional Control Scale (ATTC) and the Attention Network Test (ANT) across alerting, orienting, and executive control networks. This result aligns with previous findings questioning the validity of the ATTC as an accurate proxy for attentional control abilities. While the ATTC may reliably measure subjective perceptions of control, these metacognitive insights appear parted from actual attention regulation efficiency during behavioral testing.

Dissociations between subjective self-reports and objective performance arise due to attention’s intrinsically complex, multidimensional nature. Variability in what specific components’ different assessments emphasize can contribute to poor correlations: individual differences, limitations in self-awareness, and contrasts between testing methods and further obscure relationships.

By examining the correspondence between the ATTC and established attention control tasks, this study enhances our understanding of the conditions under which conflicting reports will arise based on assessment selections. The findings of [52,53,54,55] indicate that ATTC is not a valid measurement to assess attentional control, and using it requires more careful consideration.

Also, this work raises the question of whether a questionnaire alone is a good measure for psychological measurement. This can reconcile past correlational ATTC research contradictions through methodological and measurement dissociation. In addition, as [13,56,57] mention, we are calling for further emphasis on the need for careful consideration in using the ATTC.

The current results do not support using ATTC as a stand-alone substitute measure for attention control. A more nuanced, multimodal approach is necessary to fully capture attention’s complexities encompassing first-person experiences and third-person behaviors. Longitudinal studies tracking within-person changes and alternative measurement models may clarify boundaries between subjective self-reports and objective levels of control. Integrating multiple methods remains essential for elucidating the intricacies of attention control and advancing its assessment.

Furthermore, the reliability of questionnaires often hinges upon comparisons with preceding instruments, a point of significant importance given that the authors of [58] underline the imperative for regular estimation and disclosure of reliability in behavioral assessments within the field of psychological science. Such practices are crucial to solidify the validity of conclusions derived from statistical analyses. Similarly, the authors of [59] mention that endorsing standardized practices is essential for ensuring methodological integrity.

## 8. Limits of This Study

While providing evidence regarding the lack of correspondence between the ATTC and ANT, this study had limitations that could be addressed in future research. Our sample consisted solely of adults in a restricted age range, gathered predominantly online. The use of online surveys for data collection may face challenges such as unreliable email lists (in our case) and participant willingness. To minimize these variations, consider improving the design of search and delivery tools and engaging with variability during data preparation [60,61,62].

Further studies with larger, more diverse cohorts from broader populations and age groups are needed to generalize conclusions. We used a single self-report scale paired with one behavioral test. Employing multiple complementary subjective measures and objective tasks related to attention control would allow for a more robust evaluation of relationships. Data collection occurred wholly online, contributing to variability. Added experimental control could strengthen the reliability of the results. Although these findings question the interchangeability of the ATTC and performance indices like the ANT, continuing investigation with enhanced sampling, multimodal measurement, and tighter methodological control is vital to elucidate boundaries between perceived and actual attentional control capacities.

## Figures and Tables

**Table 1:** Table with gender changes and mean (SD) format.

Variable	Male M (SD)	Female M (SD)	*t*	*p*-Value	Cohens d	95% CI
ATTC_TOT	50.38 (4.63)	49.99 (4.79)	0.49	0.62	0.08	[−1.16, 1.93]
ATTC_FOC	22.74 (3.48)	22.07 (3.27)	1.18	0.24	0.20	[−0.44, 1.77]
ATTC_SHI	27.64 (3.48)	27.92 (3.67)	−0.46	0.64	−0.08	[−1.45, 0.90]

**Table 2:** Mean and Standard Deviation (SD) of response times.

Condition	M (SD)
Mean RT	531.353 (8.967)
Mean RT (Both)	467.11 (19.47)
Mean RT (Blank)	582.03 (10.31)
Mean RT (Center)	494.23 (19.77)
Mean RT (Congruent)	525.81 (12.95)
Mean RT (Incongruent)	570.57 (18.23)

**Table 3:** Results based on cue and warning type for all trials and correct trials.

Cue	ALL			Correct		
	Blank	Double	Center	Blank	Double	Center
Congruent	582 (39)	493 (114)	471 (99)	582 (39)	491 (114)	470 (98)
Incongruent	637 (80)	491 (118)	542 (86)	637 (81)	494 (114)	542 (85)
Neutral	560 (92)	409 (78)	496 (115)	560 (93)	409 (75)	497 (115)

**Table 4:** Table with gender changes and mean (SD) format.

Variable	Male M (SD)	Female M (SD)	*t*	*p*-Value	Cohens d	95% CI
Alerting	−114.60 (21.84)	−115.24 (21.92)	0.18	0.86	0.03	[−6.53, 7.82]
Orientation	−26.06 (25.83)	−28.20 (27.93)	0.47	0.64	0.08	[−6.69, 10.96]
Executive	−48.81 (20.22)	−40.65 (21.42)	−2.34	0.02	−0.39	[−14.99, −1.33]

**Table 5:** Correlation matrix of study variables.

	ATTC_TOT	ATTC_FOC	ATTC_SHI	IES	Alerting	Orientation	Executive
ATTC_TOT	1						
ATTC_FOC	0.65 ***	1					
ATTC_SHI	0.70 ***	−0.09	1				
IES	0.05	2.91 × 10^−3^	0.06	1			
alerting	−0.09	−0.11	−0.01	0.02	1		
orientation	−0.04	−0.04	−0.01	−0.13	0.60 ***	1	
Executive	−0.02	−0.16	0.13	0.09	0.19 *	−1.55 × 10^−3^	1

* *p* < 0.05, and *** *p* < 0.001.

## Data Availability

Data files, including the Analysis in R, detailed participants data, IES score, calculation of the ANT task, and code for calculating the ATTC questionnaire, are available at https://osf.io/rk4qa/ (accessed on 25 February 2024).
